# Nutritional Components of Wheat Based Food: Composition, Properties, and Uses

**DOI:** 10.3390/foods12214010

**Published:** 2023-11-02

**Authors:** Donatella Bianca Maria Ficco, Grazia Maria Borrelli

**Affiliations:** Consiglio per la Ricerca in Agricoltura e l’Analisi dell’Economia Agraria—Centro di Ricerca Cerealicoltura e Colture Industriali, 71122 Foggia, Italy; graziamaria.borrelli@crea.gov.it

Wheats (bread and durum wheats) and their main end-use products (particularly bread and pasta) have an important role in the Mediterranean diet as they substantially contribute to nutrient intake. Furthermore, whole grains are also a source of dietary fiber, minerals, vitamins, and phenolic compounds with beneficial effects on human health.

This Special Issue comprises a collection of ten peer-reviewed articles centered on the variability of the chemical, functional, and technological features of raw materials for the identification of those useful for the improvement of derived wheat based foods. In total, 66 authors contributed to this collection, which consists of nine research articles and one review article. Among the accepted submissions, the following topics were mainly covered: the exploitation of landraces, ancient and modern genotypes belonging to different cereal species, as a source of useful compounds to obtain staple foods with superior nutrition and functional properties (five articles); the use of functional/fortifying ingredients starting from raw materials (two articles) or by-products/wastes generated in the agribusiness industries (two articles) to improve the nutritional and antioxidant properties of bread and pasta, mainly; and finally, the optimization of the use of wheat aleurone for bakery products (one review article).

Recently, to cope with climate change and to meet the needs for more sustainable low-input grain production, the exploration of genetic diversity has become fundamental. Landraces, adapted to specific agro-climatic conditions, constitute a reservoir of genetic diversity for the development of new agricultural systems and new interesting products, with a higher content of micronutrients (minerals; vitamins) and bioactive components. In this perspective, pigmented landraces are an underexploited resource of phytochemicals. Ladhari et al. [1] investigated the compositional properties of two differently anthocyanin-rich Ethiopian durum wheat landraces in relation to protein content, dry gluten, ash, total polyphenols, anthocyanins, proanthocyanidins, and specific phenolic acids, confirming them to be a source of primary and secondary metabolites of interest for human health and nutrition. 

Furthermore, both old varieties that have been replaced with more productive ones and some of the species that were cultivated in the past have been recovered during recent decades for regional crop production and gourmet foods as they have more favorable compositions in terms of health. 

Among the ancient wheats, diploid einkorn (*T. monococcum*, L., AA genome), tetraploid emmer (*T. turgidum* L. subsp. *dicoccum* Thell., AABB genomes), and hexaploid spelt (*T. aestivum* L. subsp. *spelta* Thell, AABBDD genomes) represent interesting genetic materials. 

Lovegrove et al. [2] analyzed three cultivars of emmer and five cultivars each of spelt and bread wheat, grown in two consecutive years at two nitrogen levels that reflect those of low-input and intensive farming systems, for their dietary fiber, polar metabolites, protein, phenolics, and mineral micronutrients. While the ranges of the components resulted to be similar among the three cereal types, an exception regarding mineral micronutrients (zinc and iron) which are prevalent in spelt and emmer was found. It follows that the consumption of these wheats could contribute to the ways to cope with micronutrient deficiencies, which affect billions of people worldwide. 

The renewed interest in more natural foods promoted the use of these wheats as raw materials for the making of products such as bread, pasta, biscuits, pancakes, etc. In this regard, studies on processing quality have increased in importance. Brandolini et al. [3] studied the composition and technological characteristics of the flours and breads of two elite einkorn wheats in comparison with those of a high-quality bakery bread wheat, all cropped in four different environments. The einkorns confirmed better flour composition than that of bread wheat in terms of proteins, soluble pentosans, and yellow pigment. Technologically, they exhibit breadmaking behavior that is similar to that of bread wheat. Einkorn breads had a softer texture, are maintained for a longer shelf-life period, and have a slower retrogradation period than that of bread wheat. They demonstrate that the possibility of obtaining einkorn breads with technological properties comparable to those of breadmaking-quality bread wheat and with superior nutritional value is linked to the use of high-quality cultivars and to the optimization of processing conditions. 

In a similar approach, Huertas-García et al. [4] evaluated a wide collection (eighty-eight) of Spanish traditional spelt accessions for grain/ flour quality, dough, and baking traits and compared them with those of ten modern wheat cultivars (nine bread wheats and one modern spelt cultivar). In comparison with wheats, Spanish spelt accessions showed softer grains, higher protein content and gluten extensibility, and lower gluten strength. When considering bread-making quality, two spelt genotypes were shown to have the highest loaf volume, the most important trait through which to assess wheat baking quality, and to have the possibility to be used for breeding purposes aimed at the improvement of bread making quality. 

Finally, Borrelli et al. [5] compared old and modern cultivars of three cereal species, durum wheat, emmer wheat, and barley, in a 2-year evaluation, studying the composition of phenolics and carotenoids, and antioxidant activity in wholemeals and related biscuits, providing information on consumer acceptability. Barley showed the highest levels for all traits, followed by emmer, with the exception of carotenoid content, which was prevalent in the modern durum wheat cultivar. Generally, the highest values of phytochemicals were observed in the modern cultivars, while the old ones had the highest total phenolic content, according to the literature. The same trend was observed in biscuits. Emmer showed the best overall acceptability among consumers with even higher scores than those of commercial biscuits, while barley biscuits, albeit ranking the highest in terms of phytochemicals, were shown to be less appreciated. New insights into consumer-led food product development are required to find optimal formulations for better nutritional, sensorial, and health-related end-products. Once identified, the genetic materials deemed of interest can be recommended for supply chain studies or employed to transfer qualitative and bioactive traits into elite cultivars. 

With growing demands for functional, natural, and low-calorie ingredients, extensive research is being carried out to identify novel ingredients that can be useful in the enrichment of formulations for various cereal-based products. To this aim, the partial replacement of wheat flour with phytochemical-rich, functional plant-based flours or powders is an interesting strategy. For instance, Cornelian cherries (CC; *Cornus mas* L.) are edible fruits with a color ranging from red to purple, that are rich in vitamin C and polyphenols. CC-derived ingredients have been used in many formulations to produce enhanced food products. Šimora et al. [6] investigated cornelian cherry powder (CCP) as a functional ingredient for bread production, via the incorporation of five CCP levels (0, 1, 2, 5, and 10% *w*/*w*) in flour. Overall, the results showed that replacing wheat flour with up to 5% of CCP produced the most suitable formulation with which to yield bread loaves that are richer in antioxidants, without negatively impacting the physical properties and sensory attributes of the bakery.

In a similar way, hemp can be used to enrich functional pasta. Hemp seeds, mainly used as animal feed, are receiving great attention in relation to their use by humans as a source of nutrients, in particular for their high levels in polyunsaturated fatty acids. Bonacci et al. [7] incorporated hemp seed flour, at different percentages (5%, 7.5%, and 10%) and with two different particle sizes, as a fortifying ingredient in the production of pasta, studying the effect on its rheological and chemical characteristics. The minor particle size and 7.5% substitution level have been identified as the optimal processing conditions in which to produce cooked fortified pasta of high quality, that is nutritionally rich (with improved minerals, and an improved amino acid and polyunsaturated fatty acid content and profile), and has good organoleptic properties.

Functional ingredients may also be derived from by-products or food processing waste, with the aim of sustainably using natural resources, providing additional economic benefits to food businesses. 

An example of the circular bioeconomy comes from brewers’ spent grain (BSG), the main by-product generated from the brewing industry. BSG is rich in antioxidant compounds such as phenolic acids, flavonoids, tannins, and proanthocyanidins, amino phenolics, and fiber (β-glucans and arabinoxylans). Baiano et al. [8] considered BSG derived from the brewing of Belgian-style white beers a functional ingredient in bread making. Three different mixtures of barley malt and of unmalted bread wheat/ durum wheat/ emmer wheat, cropped in two geographical areas, were used for bread making, at two different percentages (20 and 25%) of substitution of wheat flour type 0, with the aim to evaluate the effects of such replacements on overall bread quality and functional characteristics. The authors stated that supplementation with the highest percentage of BSGs exerted a positive influence on the contents of phenolics and dietary fibers with good structural and sensory attributes, owing to the simultaneous addition of gluten. Bread supplemented with bread or durum wheat spent grains resulted in a good compromise between the content of nutraceuticals and overall sensory quality.

Further, given the growing interest of consumers in so-called “superfoods”, the use of the avocado fruit is seeing great relevance owing to its great potential, conferred by its antioxidant, fiber, and low sugar content. Avocados are mostly consumed fresh, but they can also be processed, and hence, several components of the fruit, including the peel and seeds, these being rich in protein, fiber, and numerous bioactive compounds, are wasted. This waste may constitute a suitable ingredient in the form of powders for pasta, breads, dry soups, and other food recipes. In this regard, Viola et al. [9] aimed to recycle avocado waste through dehydration and milling. The resulting powder was used as a functional ingredient for the processing of sourdough semolina bread, a product consumed daily in Southern Italy. The authors demonstrated that avocado waste products can be successfully incorporated into leavened baked products to improve their characteristics, particularly in terms of antioxidant content, also resulting in sensorially appreciation due to aroma and color. 

Owing to the rising demand for novel formulations, combining the right ingredient with an efficient processing method has become a critical endeavor. 

Although health benefits might be achieved through the use of wholemeal for cereal-based products due to its richness in bioactive compounds, it noted that the use of wholemeal, containing bran, negatively affects technological properties during food processing and the sensory acceptance of final products. A compromise could be achieved via the use of aleurone, which is considered by millers as a bran layer rich in dietary fiber, proteins, and bioactive compounds. Unfortunately, its use is limited, most likely due to issues related to its extraction and to its negative rheological and technological effects on dough, which are mainly ascribable to its content of dietary fiber, particularly the water-insoluble type. Therefore, specific modifications to aleurone’s components, performed without losing any of the health benefits prior to its incorporation into a food process, are welcomed. 

Lebert et al. [10] reviewed different extraction techniques to test the potentiality of this tissue as a nutrient-rich ingredient in bread-making. The authors found an improved nutritional profile in aleurone-rich flour including dietary fiber and protein, minerals, antioxidants, phytoestrogens, and sterols. Despite these beneficial nutritional properties, changes during dough development could affect pasting properties and dough rheology, depending on the percentage of aleurone in the flour. Considering that the aleurone layer is tightly bound to seed coats, it is rather difficult to separate it from the endosperm and from the rest of the bran during conventional milling. Most existing processes for the retrieval of the aleurone layer begin with bran material and provide for the main mechanical, physical and chemical procedures, with varying aleurone purities and yields. Moreover, reducing the particle size of an aleurone material can also lead to beneficial effects in terms of the bioaccessibility of antioxidant compounds. Unfortunately, there is no an univocal method related to its extraction, and the latter also depends on the natural variability of the grain’s constituents, constantly requiring the adaptation of the process itself to each raw material. Further, for the wide use of aleurone as an ingredient, extraction methods should be standardized, easy to apply, and not too costly.

The articles compiled in this Special Issue demonstrate a research trajectory in which natural variation in the grain composition of wheats and related cereals, the use of functional food ingredients, and the process used should be considered when studying the wheat supply chain.
